# Bio-priming with a hypovirulent phytopathogenic fungus enhances the connection and strength of microbial interaction network in rapeseed

**DOI:** 10.1038/s41522-020-00157-5

**Published:** 2020-10-30

**Authors:** Zheng Qu, Huizhang Zhao, Hongxiang Zhang, Qianqian Wang, Yao Yao, Jiasen Cheng, Yang Lin, Jiatao Xie, Yanping Fu, Daohong Jiang

**Affiliations:** 1 State Key Laboratory of Agricultural Microbiology, Huazhong Agricultural University, Wuhan, 430070 Hubei Province China; 2 Hubei Key Laboratory of Plant Pathology, College of Plant Science and Technology, Huazhong Agricultural University, Wuhan, 430070 Hubei Province China

**Keywords:** Applied microbiology, Next-generation sequencing

## Abstract

Plant disease is one of the most important causes of crop losses worldwide. The effective control of plant disease is related to food security. *Sclerotinia* stem rot (SSR) caused by *Sclerotinia sclerotiorum* leads to serious yield losses in rapeseed (*Brassica napus*) production. Hypovirulent strain DT-8 of *S. sclerotiorum*, infected with Sclerotinia sclerotiorum hypovirulence-associated DNA virus 1 (SsHADV-1), has the potential to control SSR. In this study, we found rapeseed bio-priming with strain DT-8 could significantly decrease the disease severity of SSR and increase yield in the field. After bio-priming, strain DT-8 could be detected on the aerial part of the rapeseed plant. By 16S rRNA gene and internal transcribed spacer (ITS) sequencing technique, the microbiome on different parts of the SSR lesion on bioprimed and non-bioprimed rapeseed stem was determined. The results indicated that SSR and bio-priming treatment could influence the structure and composition of fungal and bacterial communities. Bio-priming treatment could reduce the total abundance of possible plant pathogens and enhance the connectivity and robustness of the interaction network at the genus level. This might be one of the mechanisms that rapeseed bioprimed with strain DT-8 had excellent tolerance on SSR. It might be another possible mechanism of biocontrol and will provide a theoretical guide for agricultural practical production.

## Introduction

Based on the estimate of the United Nations, the global population will reach 9.7 billion by mid-century and 10.8 billion by the end of this century^1^. Food production needs to rise by 70% roughly by 2050 and double or triple by 2100^2^. It is a great challenge to human beings. To fulfill the request, on one hand, we should improve the yield and creating efficient cultivation techniques; on the other hand, we should protect plants from pests and diseases^3^.

Rapeseed (*Brassica napus* L.) is an important oil-producing plant in the world and the second large cultivated oilseed crop next to soybean^4^. Concurrently, *Sclerotinia* stem rot (SSR) caused by the necrotrophic fungal pathogen *Sclerotinia sclerotiorum* (Lib.) de Bary is a major threat of rapeseed^5^ and always causes devastating yield losses^6^. When a rapeseed plant is infected, as the necrotic lesions girdle the stem and cause the stem to lose its rigidity, the major cause of SSR-induced yield loss, lodging, may occur^7^. Due to the lack of effective resistant cultivars, the control of the disease mainly depends on chemical fungicides^7^. Considering the development of the fungicide-resistant population of *S. sclerotiorum* and environmental problems, it is important to explore novel and environmentally friendly alternatives or to reduce the amount of chemical fungicides used^8^.

Mycoviruses or fungal viruses are viruses that infect fungi persistently^9^. Usually, mycoviruses do not affect the phenotype of hosts. However, some mycoviruses can cause amazing changes in their hosts, including irregular growth, abnormal pigmentation, altered sexual reproduction, and hypovirulence^9–12^, and therefore have the potential to control plant fungal diseases^9^. Sclerotinia sclerotiorum hypovirulence-associated DNA virus 1 (SsHADV-1) is a circular single-stranded DNA virus originally isolated from the hypovirulent *S. sclerotiorum* strain DT-8^13^. SsHADV-1 can infect its fungal host extracellularly and drive a mycophagous insect, *Lycoriella ingenua* as a transmission vector^14^. The SsHADV-1-infected *S. sclerotiorum* strain has enormous potential as a biological control agent (BCA)^15^.

Biological priming (bio-priming) is an advantageous technique that incorporates biological inoculation of seed with beneficial microorganisms to guard seeds and regulate seed hydration for abiotic and biotic stress management^16^. Maize seed bioprimed with *Trichoderma lixii* ID11D could alleviate salt toxicity and increased the lengths, fresh and dry weights of the root and shoots^17^. *Thalassobacillus denorans* NCCP-58 and *Oceanobacillus kapialis* NCCP-76 enhanced the growth of rice under different salinity concentrations when applied through bio-priming^18^. Tomato plants primed with *Trichoderma pseudokoningii* BHUR2 were healthier and the anti-oxidative enzyme activity was augmented upon challenge with *Sclerotium rolfsii*^19^. Increased germination and seedling vigor and decreased disease incidence were observed in durum wheat after primed with rhizospheric and endophytic bacteria^20^. Through seed bio-priming, the beneficial microorganisms can occupy the growing root surfaces, form a biofilm around the roots, and protect the plants from soil-borne plant pathogens. At the same time, seed bio-priming can protect the plants from foliar pathogens by eliciting systemic resistance of plants during all the stages of their growth and development. Seed bio-priming can also enhance nutritional and physiological characteristics and result in better germination and adaptation under different soil conditions^21^.

The microbiome is defined as the whole microorganisms and their genomes in a special living environment^22^. The microbiome of plants, including phyllosphere microorganisms, rhizosphere microorganisms, and endogenous microorganisms, plays a crucial role in both plant and ecosystem health^23^. The microbiome of healthy plants protects them from the harm of pathogens, thus establishs and encourages a ‘healthy microbiome’ to control plant diseases and improve the yield^24^. The microbiome is based on the multi-interaction among host plants, pathogens, BCAs, and other microbial communities^25^. Schmidt et al.^26^ found that the structure of rhizosphere bacterial communities of chamomile plants was significantly changed after treatment with beneficial bacteria. Sylla et al.^27^ clarified that Trianum-P (*Trichoderma harzianum* T22) treatment could change the fungal community’s composition and diversity of strawberry phyllosphere. However, it is unclear how the structure and diversity of plant microbiome changed after bio-priming, and the internal relationship between the microbiome change and the increased abiotic and biotic tolerance.

SSR is a serious threat to rapeseed. In this study, the mycovirus-mediated hypovirulent strain DT-8 was applied to bio-prime the rapeseeds to control SSR. Through the field experiment, we found the bioprimed rapeseed could suppress SSR and increase rapeseed yield significantly, and the effects were similar to that of chemical fungicide (prochloraz) treatment. This suggested that bio-priming treatment might provide the possibility to decrease the usage of chemical pesticides for controlling SSR. Besides the possible direct effects on the virulence of *S. sclerotiorum* and the possible effects on rapeseed resistance, we assumed the bio-priming treatment could also influence the diseased stem microbiome of rapeseed to enhance the tolerance to SSR. To prove this hypothesis, the microbiome of SSR lesions on the bioprimed and non-bioprimed rapeseed stem was analyzed by the 16 S rRNA and internal transcribed spacer (ITS) sequencing. The relationship between symptoms of SSR and microbiome, and the impacts of biopriming treatment on plant microbiome were explored.

## Results

### The effect of bio-priming treatment in the field experiment

**Fig. 1:**
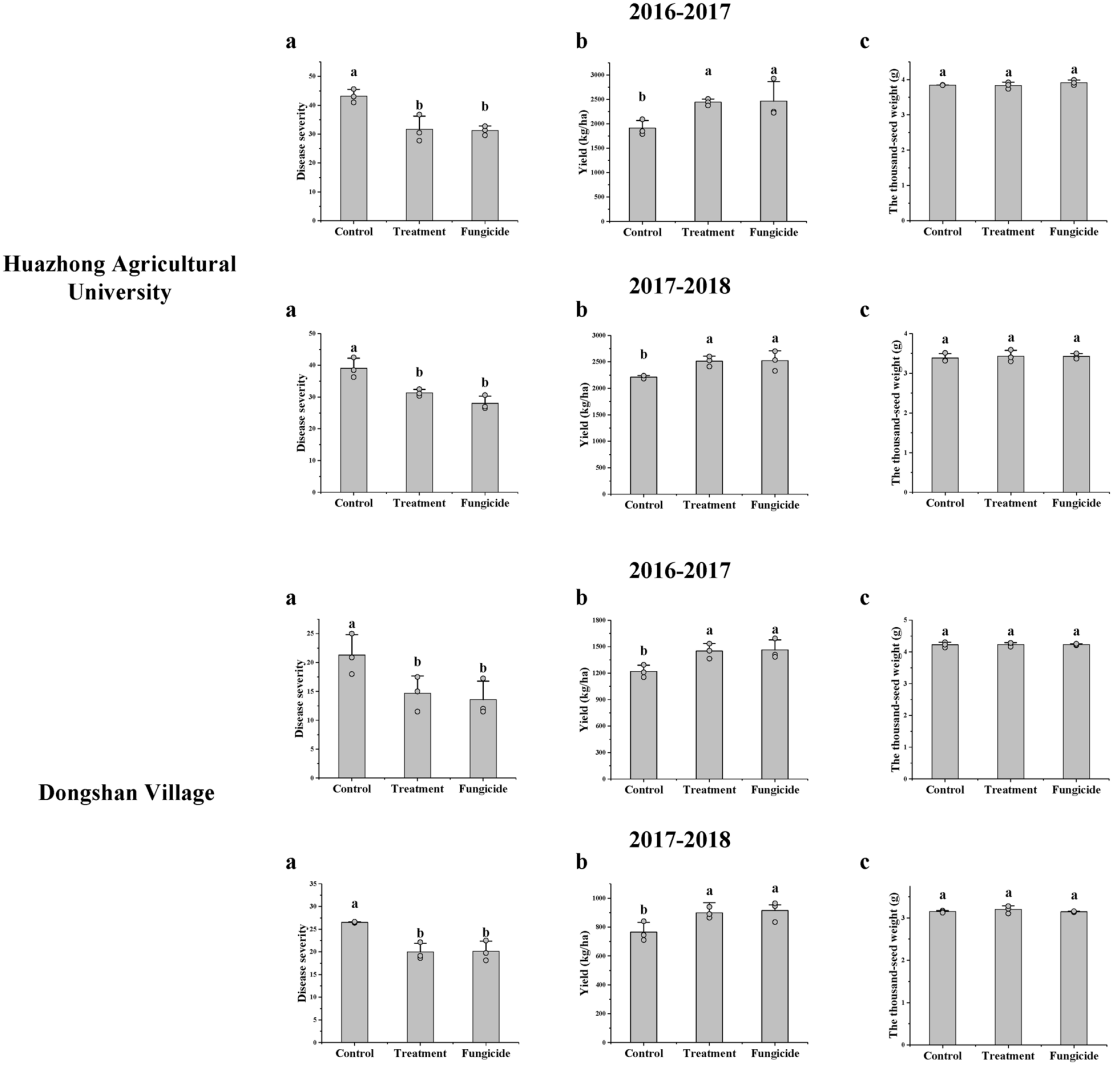
The field experiments. **a** The disease severity of SSR. **b** The yield. **c** The thousand-seed weight. Data were analyzed by one-way ANOVA followed by DMRT. Means for each index followed by different letters are significantly different at *P* < 0.05. The error bars represent the SEMs. Control: Non-bioprimed rapeseed. Treatment: Bioprimed rapeseed. Fungicide: The rapeseed were sprayed with fungicide prochloraz (150 g a.i./ha) at the flowering stage.

Through the field experiment, we found the tolerance of bioprimed rapeseed for SSR was increased. During 2016–2018, in Huazhong Agricultural University and Dongshan Village, bio-priming treatment could significantly decrease the disease severity of SSR and increase the yield (Fig. 1a, b). No treatment had significant effects on the thousand-seed weight (Fig. 1c). The control of SSR and the increase of the yield by bio-priming treatment were similar to the chemical control prochloraz. Compared to spraying chemical fungicide (prochloraz) (mean 29.05 ± 5.12% and 20.63 ± 6.28%, respectively), bio-priming treatment could reduce mean 25.6 ± 4.68% the disease severity of SSR and largely increase the seed yield by mean 19.43 ± 6.01%.

### PCR detection of SsHADV-1

For the natural diseased rapeseed stem of the bio-priming treatment collected from the field, coat protein gene (*CP*) of SsHADV-1 was detected in some of the samples taken from the bioprimed treatment, whereas it could not be detected in any samples of the non-bioprimed control (Supplementary Fig. 1). The results indicated that *S. sclerotiorum* DT-8 could colonize the aerial part of the rapeseed plant in the field after bio-priming.

### Overview of all sequencing data and taxonomy assignments

For all sequencing data of 16S rRNA gene, after quality trimming and chimera checking, a total of 1,202,872 reads were obtained. After removing no-target amplicon sequence variants (ASVs) (14,845 reads) and low-abundance ASVs (475 reads), 1,187,552 high-quality processed sequences were obtained with a median read count per sample of 49313.5 (range: 34,159–58,491). This corresponded to a total of 1230 ASVs. Referring to the rarefaction curves (Supplementary Fig. 2a), the dataset was normalized to the lowest number of read counts (34,159 reads). After taxonomy assignments, the 1230 ASVs were classified into 223 genera in 50 orders and 12 phyla. *Proteobacteria* (59.87%), *Actinobacteria* (30.82%), and *Bacteroidetes* (8.59%) were the top three phyla (Supplementary Figs 3b and 4a).

For all sequencing data of ITS, after quality trimming and chimera checking, a total of 1,127,608 reads were obtained. After removing the no-target ASVs (503 reads) and low-abundance ASVs (31 reads), 1,127,074 high-quality processed sequences were obtained with a median read count per sample of 46,635 (range: 38,801–54,861). This corresponded to a total of 147 ASVs. Referring to the rarefaction curves (Supplementary Fig. 2b), the dataset was normalized to the lowest number of read counts (38,801 reads). After taxonomy assignments, the 147 ASVs were classified into 44 genera in 22 orders and 2 phyla. *Ascomycota* (69.06%) was the most abundant followed by *Basidiomycota* (30.94%) (Supplementary Figs 3b and 4c).

### Diversity analysis of bacterial and fungal communities

**Fig. 2:**
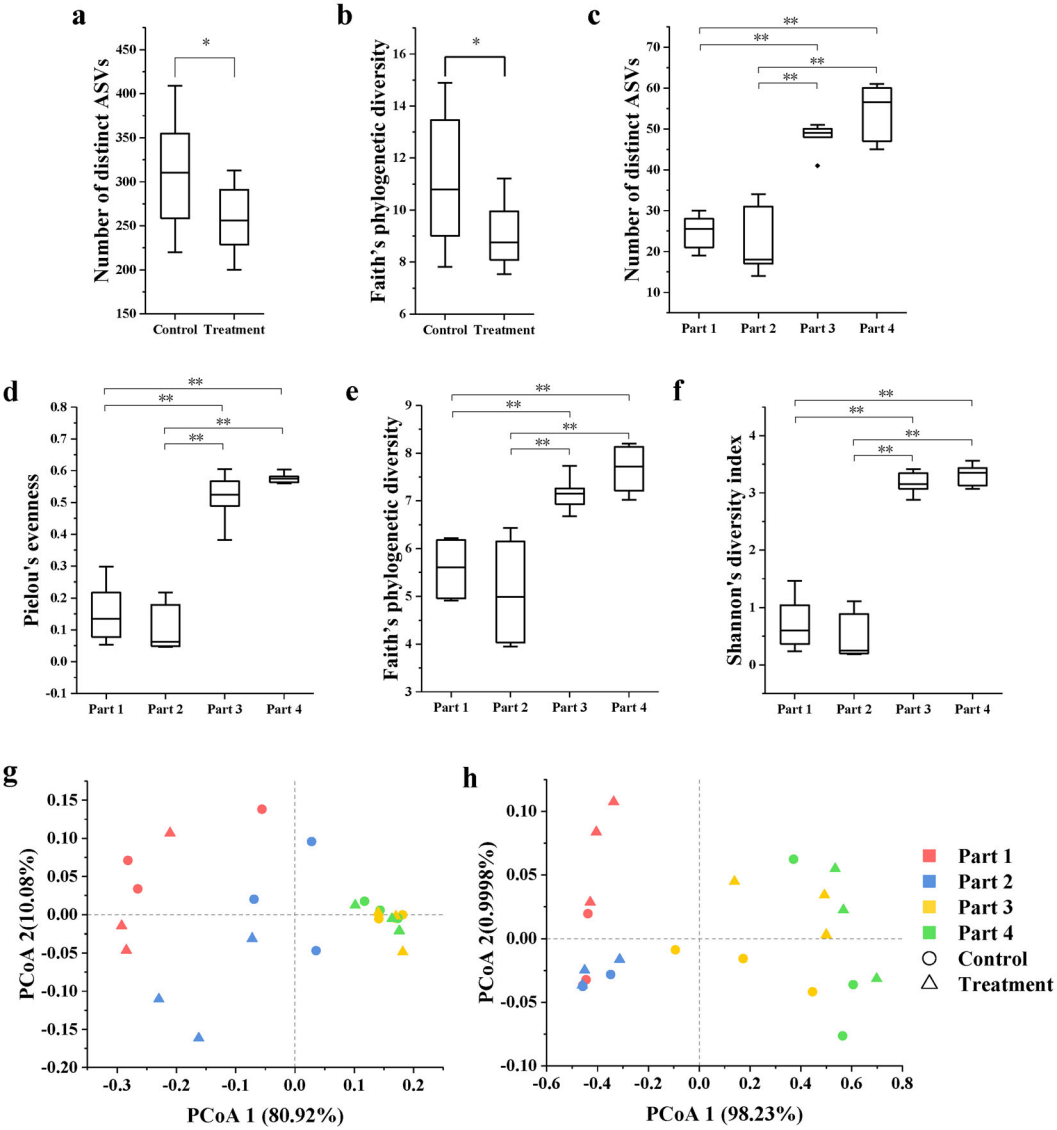
The α- and β-diversity of bacterial and fungal communities. **a** Number of distinct ASVs of bacterial communities in the treatment group and the control group. **b** The Faith’s phylogenetic diversity of bacterial communities in the treatment group and the control group. **c** Number of distinct ASVs of fungal communities in different parts. **d** The Pielous’s evenness of fungal communities in different parts. **e** The Faith’s phylogenetic diversity of fungal communities in different parts. **f** The Shannon’s diversity index of fungal communities in different parts. **g** The PCoA based on the weighted UniFrac distance of bacterial communities. **h** The PCoA based on the weighted UniFrac distance of fungal communities. The Kruskal–Wallis test was used to analyze the statistical differences in α-diversity. Levels of significance: *q-value < 0.05, **q-value < 0.01. Control: Non-bioprimed rapeseed. Treatment: Bioprimed rapeseed. Part 1: The center of the lesion. Part 2: The edge of lesion. Part 3: The healthy tissue 1. Part 4: The healthy tissue 2.

For α-diversity analysis, the numbers of ASVs, Pielous’s evenness, Shannon’s diversity index, and Faith’s phylogenetic diversity were used to evaluate the richness, evenness, and diversity of bacterial communities and fungal communities. For bacterial communities, compared with the control group, the number of ASVs and Faith’s phylogenetic diversity of the treatment group were significantly lower (Fig. 2a, b), but the Pielous’s evenness and Shannon’s diversity index were not significantly different (Supplementary Fig. 5c, f), whereas there were no significant differences between the healthy and diseased lesion tissues (Supplementary Fig. 5a, b, d, e). For fungal communities, there was no significant difference between the control group and the treatment group (Supplementary Fig. 5g–j). However, all α-diversity indices of the healthy lesion tissues were significantly higher than those of the diseased lesion tissues (Fig. 2c–f). The results showed that bio-priming could significantly decrease the richness and Faith’s phylogenetic diversity of bacterial communities, and SSR could significantly decrease the richness, evenness, and diversity of fungal communities.

The principal coordinate analyses (PCoA) based on the weighted UniFrac distance was used for β-diversity analysis. The first two principal coordinates of the β-diversity of bacterial and fungal communities accounted for 91.00% and 99.23% of the total variation, respectively. The major driver of β-diversity of bacterial and fungal communities was different tissues of the lesion (accounting for 80.92% and 98.23% of the total variance), followed by the bio-priming treatment (accounting for 10.08% and 0.9998% of the total variance). The PCoA also showed the bacterial communities distinctly clustered on the healthy group (Fig. 2g). Except for Part 3 (the healthy tissue 1) and Part 4 (The healthy tissue 2), there were significant differences between any two parts (Supplementary Table 1). Compared with Part 3 and Part 4, the fungal communities of Part 1 (the center of the lesion) and Part 2 (the edge of lesion) were similar (Fig. 2h). The permutational multivariate analysis of variance (PERMANOVA) showed that there was no significant difference only between Part 1 and Part 2 (Supplementary Table 1). Both bacterial and fungal samples separating across the first principal coordinate indicated that the largest source of variation in microbial communities on rapeseed stem infected by *S. sclerotiorum* was different tissues of the lesion rather than the bio-priming treatment.

### Composition of bacterial and fungal communities

In bacterial communities, *Proteobacteria* was the dominant phylum in all sample groups. The relative abundance of *Proteobacteria* was higher in the treatment group than in the control group in each part but lower in the healthy group than in the diseased group. However, the relative abundance of *Actinobacteria* in the treatment group was lower than in the control group, while greater in the healthy group than in the diseased group (Supplementary Fig. 4a).

**Fig. 3:**
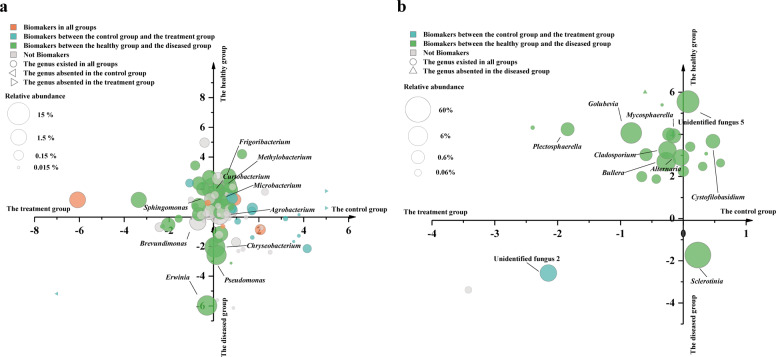
The composition of bacterial and fungal communities on lesions of rapeseed. **a** The bacterial communities. **b** The fungal communities. Only the genera of high relative abundance (over 0.01%) were shown. Different colors represent different biomakers. The size of geometry shows the relative abundance of genera. The *x*-axis shows the logarithm of the ratio of relative abundance in the control group vs. the treatment group and the *y*-axis shows a logarithm of the ratio of relative abundance in the healthy group vs. the diseased group.

There were 87 genera of which total relative abundance was over 0.01% in bacterial communities. The relative abundance of 34 genera in the diseased group was higher than in the healthy group. The relative abundance of 27 genera in the treatment group was higher than in the control group (Fig. 3a). The top ten genera in all sample groups were *Agrobacterium*, *Brevundimonas*, *Chryseobacterium*, *Curtobacterium*, *Erwinia*, *Frigoribacterium*, *Methylobacterium*, *Microbacterium*, *Pseudomonas*, and *Sphingomonas*. Only the relative abundance of *Chryseobacterium*, *Pseudomonas*, and *Erwinia* in the diseased groups was higher than in the healthy group. Only the relative abundance of *Brevundimona*s, *Erwinia*, and *Sphingomonas* in the treatment group was higher than in the control group (Fig. 3a and Supplementary Fig. 4b). Linear discriminant analysis effect-size (LEfSe) analysis revealed 22 and 17 biomakers in the healthy and diseased groups with 16 and 4 biomakers in the control and the treatment groups, respectively (Fig. 3a and Supplementary Fig. 6a, b). It indicated that both SSR and the bio-priming treatment could reduce the relative abundance of most bacterial genera.

In fungal communities, *Ascomycota* was the dominant phylum in the diseased group and the relative abundance of *Ascomycota* reduced in the healthy group (Supplementary Fig. 4c). There were 24 genera of which the total relative abundance was over 0.01% in fungal communities. The relative abundance of 3 genera in the diseased group was higher than in the healthy group. The relative abundance of 16 genera in the treatment group was higher than in the control group (Fig. 3b). The top ten genera in all sample groups were *Alternaria*, *Bullera*, *Cladosporium*, *Cystofilobasidium*, *Golubevia*, *Mycosphaerella*, *Plectosphaerella*, *Sclerotinia*, unidentified fungus 5, and unidentified fungus 2. Unidentified fungus 5 belonged to *Entylomatales* and unidentified fungus 2 belonged to *Hypocreales*. As the pathogen of SSR, *Sclerotinia* was the dominant genus in the diseased group. Interestingly, the relative abundance of *Sclerotinia* was also high (12.80% ~ 47.12%) in the healthy group. The treatment group had a lower relative abundance of *Sclerotinia* compared to the control group (Fig. 3b and Supplementary Fig. 4d). LEfSe analysis revealed 21 and 1 biomakers in the healthy and diseased groups with 0 and 1 biomakers in the control and treatment group, respectively (Fig. 3b and Supplementary Fig. 6c, d). It showed that SSR had an adverse impact on most fungal genera, whereas the bio-priming treatment did not.

### Composition of possible plant pathogens

**Fig. 4:**
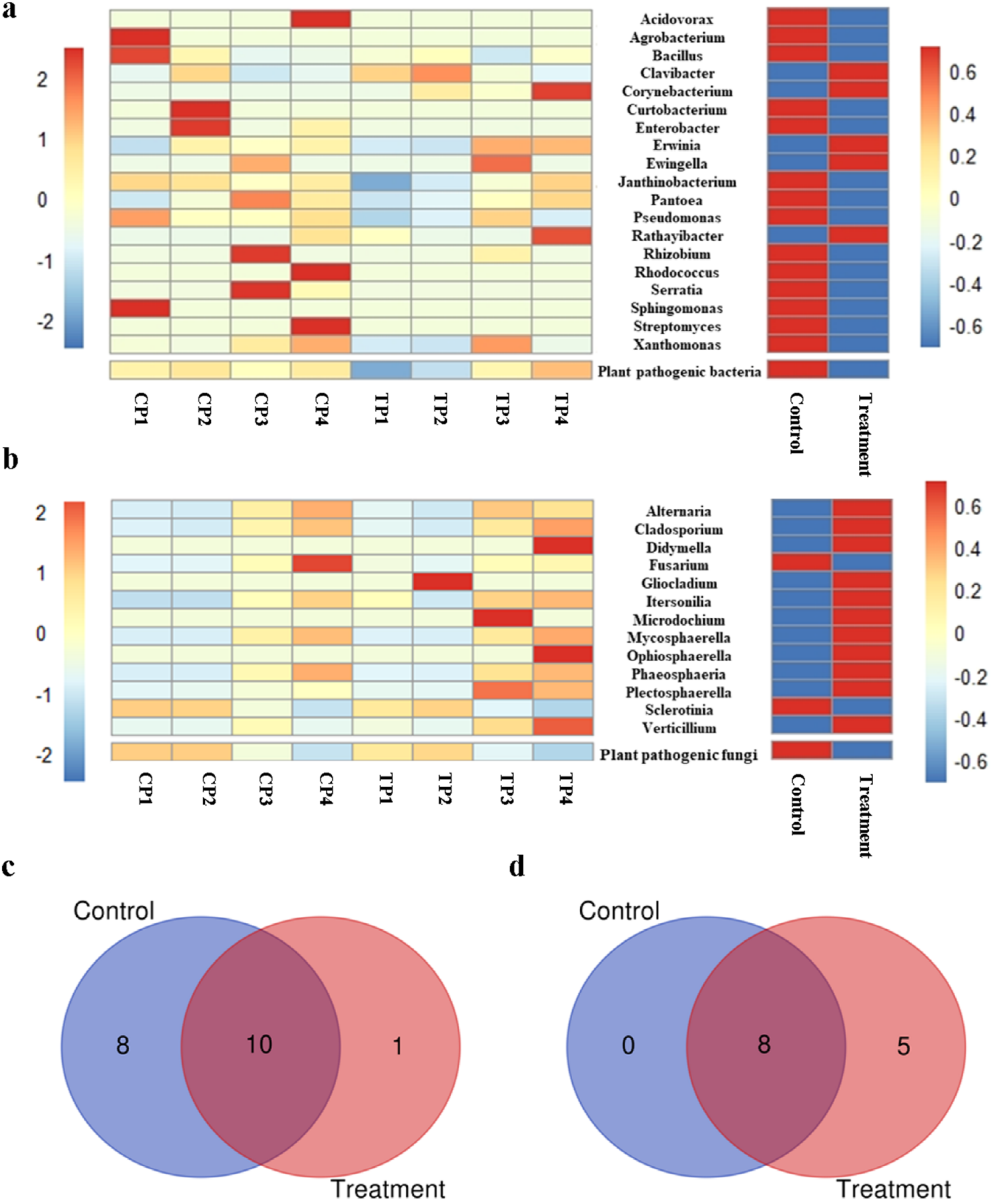
The possible plant pathogens at the genus level. **a** The possible plant pathogenic bacteria. **b** The possible plant pathogenic fungi. The common and unique possible plant pathogenic bacteria (**c**) and fungi (**d)** among the control and treatment. Control: Non-bioprimed rapeseed. Treatment: Bioprimed rapeseed. CP1: The center of the lesion of non-bioprimed rapeseed. CP2: The edge of lesion of non-bioprimed rapeseed. CP3: The healthy tissue 1 of non-bioprimed rapeseed. CP4: The healthy tissue 2 of non-bioprimed rapeseed. TP1: The center of the lesion of bioprimed rapeseed. TP2: The edge of lesion of bioprimed rapeseed. TP3: The healthy tissue 1 of bioprimed rapeseed. TP4: The healthy tissue 2 of bioprimed rapeseed.

From the data, 19 possible plant pathogenic bacterial genera and 13 possible plant pathogenic fungal genera were identified (Fig. 4a, b). Among the possible plant pathogens, there were five possible rapeseed pathogenic bacteria and seven possible rapeseed pathogenic fungi at genus level (Supplementary Table 3). There were ten common genera of possible plant pathogenic bacteria among the control and treatment group (Fig. 4c). The total abundance of all possible plant pathogenic bacteria and varieties of the possible plant pathogenic bacteria decreased after bio-priming. Except for *Clavibacter*, *Corynebacterium*, *Erwinia*, *Ewingella*, and *Rathayibacter*, the possible plant pathogenic bacteria in the control group were more abundant than in the treatment group (Fig. 4a). Among the possible plant pathogenic fungi, there were 13 genera in the treatment group, of which 8 were also detected in the control group (Fig. 4d). Compared to the control, the total abundance of all possible plant pathogenic fungi in the treatment group decreased, whereas the varieties increased. Only the abundance of *Fusarium* and *Sclerotinia* in the treatment group was lower than in the control group (Fig. 4b). The results showed that bio-priming treatment could decrease the abundance of possible plant pathogens, but increase the varieties of possible plant pathogenic fungi. Due to technical limitations of the 16S rRNA gene and ITS amplicon sequencing and the diversity of species within the same genus, the function prediction based on taxon at genus level is deficient. However, it still had some significance in both theory and practice.

### Microbial interaction networks in the rapeseed stem infected by *S. sclerotiorum*

One hundred and six genera in the control and 97 genera in the treatment groups were selected to construct the networks. Following the random matrix theory (RMT)-based network analysis method with the threshold 0.87, a network was constructed for the control group (80 nodes) and 1 for the treatment group (78 nodes). Compared to the control group (157), the network of the treatment group had fewer nodes but more links (201) (Table 1).

**Table 1:** Topological property of the empirical networks of microbial communities.

Condition	No. of original genera	Similarity threshold (*s*_t_)	Nodes	Links	Positive links	Negative links	*R*^2^ of power law	Modularity (no. of modules)	Small-world coefficient	Natural connectivity	Average connectivity	Average path distance	Average clustering coefficient
Control	106	0.87	80	157	103 (65.61%)	54 (34.39%)	0.852	0.555^*^ (8)	2.605	3.511	3.925	3.541^*^	0.221
Treatment	97	0.87	78	201	128 (63.68%)	73 (36.32%)	0.808	0.539 (4)	2.133	6.254	5.154	3.452	0.304^*^

The Student’s *t*-test was employed to test the statistical differences of network indices using the SDs derived from corresponding random networks. Levels of significance: *P < 0.01.

**Fig. 5:**
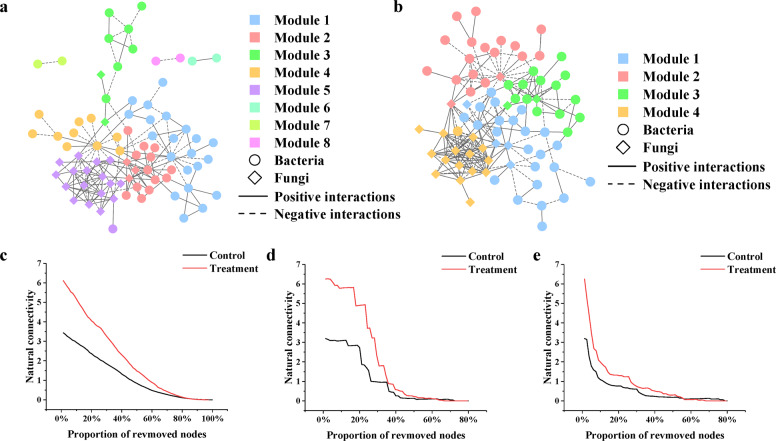
The interaction network analysis of microbiome in rapeseed stems infected by *S. sclerotiorum*. **a** The microbial interaction network of the control group. **b** The microbial interaction network of the treatment group. **c** Random attack. **d** Targeted attack ordered by betweenness. **e** Targeted attack ordered by node degree. Control: Non-bioprimed rapeseed. Treatment: Bioprimed rapeseed.

As the *R*^2^ of power law of two networks was not <0.8, the two networks appeared to be scale free. Compared to the random networks, the average clustering coefficient of the two empirical networks was much bigger, while the average path distance was similar. The small-world coefficients were greater than 1.0. Therefore, the two networks had the small-world character. The modularity was >0.4 so that the two networks were modular (Table 1 and Supplementary Table 2). The network of the control group had eight modules and four modules in the network of the treatment group (Table 1 and Fig. 5a, b). The natural connectivity of the treatment group network was higher than that of the control group (Table 1). In the random attack setting, the natural connectivity of the treatment group network was consistently higher than the control group network (Fig. 5c). In the betweenness and node degree-based attack schemes, the treatment group network was more robust than the control group network before removing 62% and 55% nodes of each network (Fig. 5d, e). The results illustrated that the robustness of the treatment group was stronger than the control group.

**Fig. 6:**
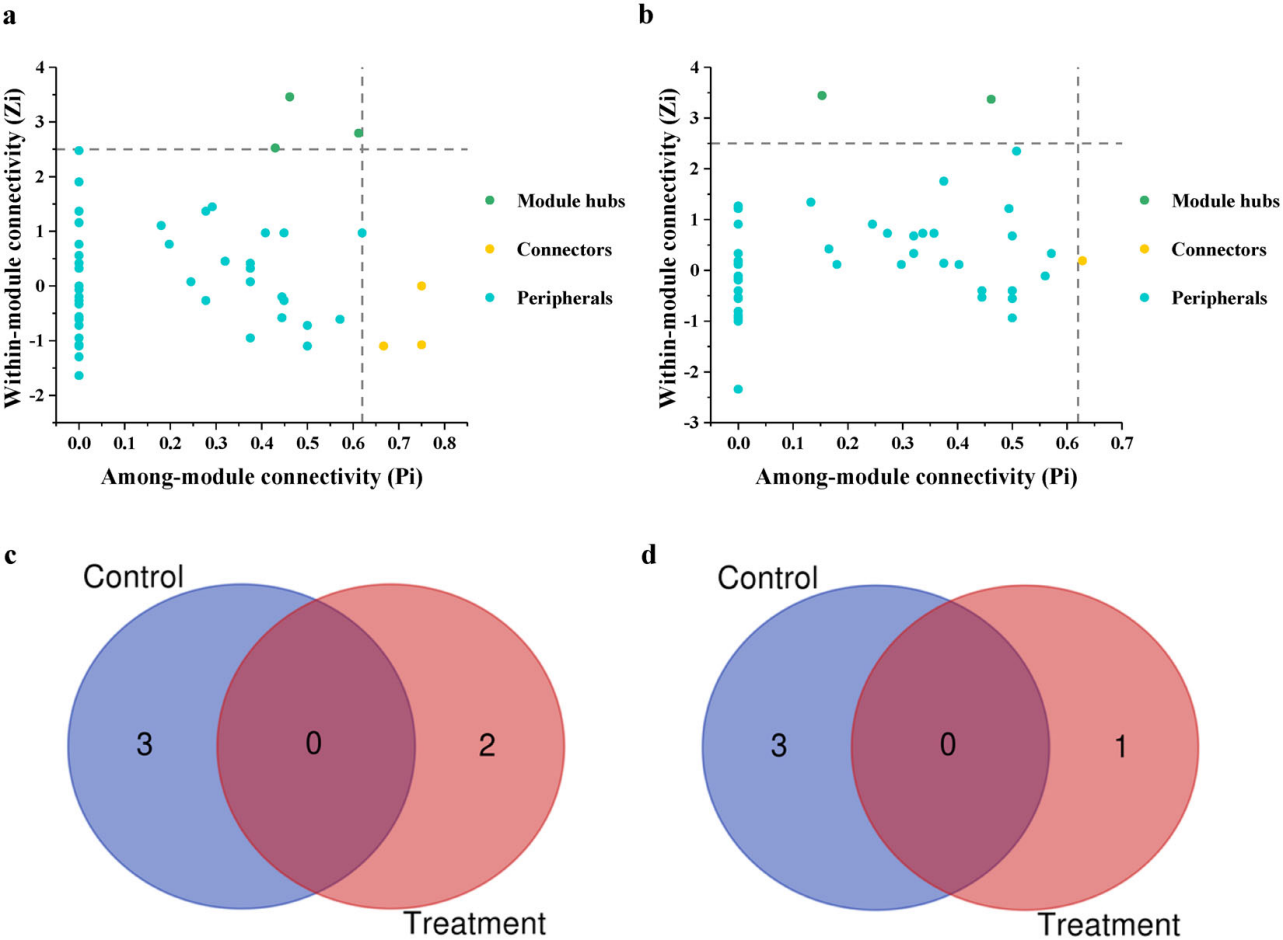
The topological roles of the genera identified in networks. **a** The topological roles of nodes for the control group. **b** The topological roles of nodes for the treatment group. **c** The common and unique module hubs between the control and treatment groups. **d** The common and unique connectors between the control and treatment groups. Control: Non-bioprimed rapeseed. Treatment: Bioprimed rapeseed.

The topological roles of the genera identified in these two networks are shown in Fig. 6a, b. The majority of the genera (92.50% for the control group and 96.15% for the treatment group) were peripheral nodes with most of their links inside their modules. Among these peripheral nodes in the control group, 60.00% had no links at all outside their own modules (i.e., *Pi* = 0), which was higher than in the treatment group (56.41%). For the control group, 7.50% genera were generalists, including 3.75% module hubs and 3.75% connectors. For the treatment group, 2.56% genera belonged to module hubs and 1.28% belonged to connectors. Between the control group and treatment group, there were no common module hubs and connectors (Fig. 6c, d). The results indicated that bio-priming treatment could change the amount and the kind of keystone microorganisms.

Thirteen genera had a negative interaction with *Sclerotinia* in the control group and treatment group (Supplementary Fig. 7). *Alternaria*, *Bullera*, *Cladosporium*, *Cryptococcus*, *Cystofilobasidium*, *Epicoccum*, *Filobasidium*, *Golubevia*, *Mycosphaerella*, *Phaeosphaeria*, and unidentified fungus 5 were common among the treatment and control group. They were the key microorganisms interacting with *Sclerotinia* and might become new BCAs.

### Phylogenetic analysis of the key microorganisms interacting with *Sclerotinia*

**Fig. 7:**
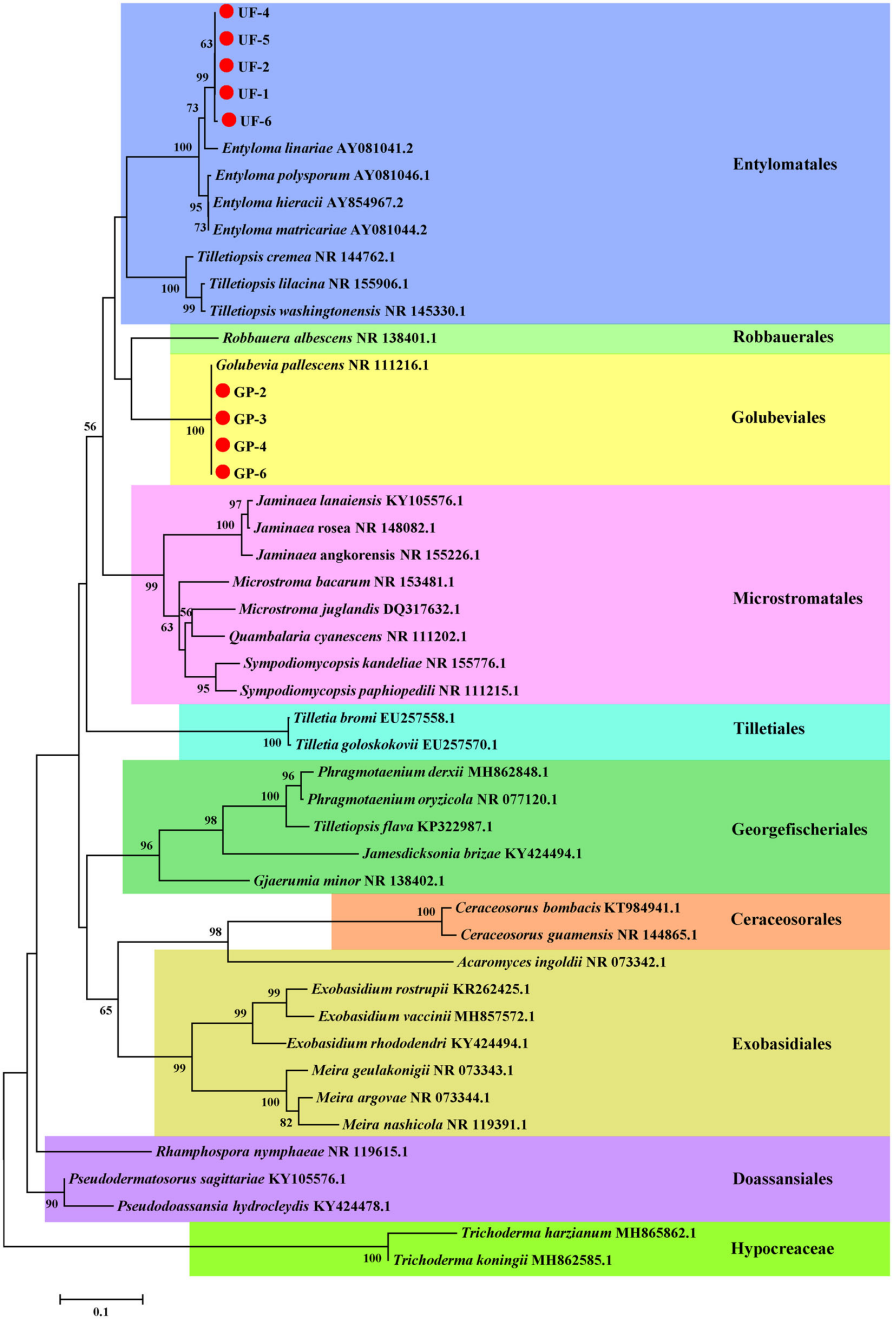
Phylogenetic analysis of the key microorganisms interacting with Sclerotinia. Phylogenetic tree were constructed by the neighbor-joining method with a bootstrap value of 1,000 replicates on ITS in MEGA 7.0.26. Bootstrap values ≥ 50% are marked above the branches.

To identify the key microorganisms at the species level and confirm the authenticity of the sequencing data, the two highly abundant key microorganisms that had a negative interaction with *Sclerotinia* were identified by TA cloning. We successfully obtained the partial ITS sequences of *Golubevia* (GP-2, GP-3, GP-4, and GP-6) and unidentified fungus 5 (UF-1, UF-2, UF-4, UF-5, and UF-6). The phylogenic analysis showed that GP-2, GP-3, GP-4, and GP-6 clustered with ITS sequence of *Golubevia pallescens*, whereas UF-1, UF-2, UF-4, UF-5, and UF-6 were closely related to that of *Entyloma linariae* (Fig. 7).

## Discussion

In general, plant diseases reduce crop yields by 10–20%^23^. It’s a worldwide problem to prevent the economic losses caused by plant diseases. In this study, we devised a way to use the virus-mediated hypovirulent strain *S. sclerotiorum* DT-8 to control SSR and increase the yield by rapeseed bio-priming. After bio-priming, strain DT-8 could successfully colonize the above-ground part of rapeseed. By 16S rRNA gene and ITS-sequencing techniques, we found bio-priming and SSR could impact the composition and structure of microbial communities. Bio-priming could also decrease the total abundance of possible plant pathogens. Through analysis of microbial interaction network, bio-priming could improve the connectivity and robustness of network at the genus level, which may be one of the key reasons for rapeseed bio-priming with *S. sclerotiorum* DT-8 to suppress SSR and increase the yield.

Biological control using living organisms to control pests is a good choice for crop protection and an important alternative of chemical control^28^. As one of the delivery strategies of biological control, seeds bio-priming has already been available, especially to control the soil-borne diseases. Seeds of Faba bean (*Viciafabae*) bioprimed with many antagonistic fungal and bacterial agents could control root rot with a long-term activity^29^. Seed bio-priming also can help control airborne diseases. Bio-priming with *P. fluorescens* induced resistance in pearl millet (*Pennisetum glaucum* [L.] R. Br) against downy mildew (*Sclerospora graminicola*) and increased the yield^30^. In this study, bio-priming with *S. sclerotiorum* strain DT-8 significantly decreased the disease severity of SSR and increased the yield in the field. It is another successful case of using seed bio-priming to control airborne diseases.

For biological management of plant diseases, successful colonization of BCAs is a key factor^31^. Detection of *CP* gene from SsHADV-1 in the bioprimed samples indicated that strain DT-8 could successfully colonize the above-ground part of rapeseed by bio-priming. The transmission of mycoviruses from viral hypovirulent fungal strains to virulent fungal strains is one of the most important advantages of using mycoviruses to control crop diseases^32^. SsHADV-1 reduces virulence and inhibits the production of sclerotia in *S. sclerotiorum*^13^ and hence decreases the source of primary infection in SSR^33^. Therefore, rapeseed bio-priming with *S. sclerotiorum* DT-8 might have a long-term control effect on SSR.

In this study, we found rapeseeds bioprimed with *S. sclerotiorum* DT-8 could drive down the richness and Faith’s phylogenetic diversity of bacterial communities, but could increase the abundance of most high-abundance fungi. For fungal BCAs, the induced systemic resistance (ISR) is one of the mechanisms to control plant diseases^34^ and also influence the composition and structure of plant microbiome. For bacterial communities in the phyllosphere, compared to the wild-type *Arabidopsis thaliana* Col-0, higher population densities of cultivable bacteria were found in mutants *etr1* and *npr1*, which were defective in ISR^35^. On the one hand, after the bio-priming treatment, the ISR of rapeseed plant might be activated by successful colonization of *S. sclerotiorum* DT-8 and might change the composition and structure of microbiome. On the other hand, the hypovirulence mediated by SsHADV-1 could weaken the ability for survival and reproduction of *S. sclerotiorum*, and the lower the abundance of *Sclerotinia* might allow for growth of other fungi.

Nowadays, the major methods of plant disease management are to control pathogens directly and to activate resistance of plants^36^. However, microbial diversity and balance are also identified as a key factor for enhancing plant health and controlling plant diseases^37,38^. Changes in the composition of the microbiome are frequently associated with infection and disease^39^. Ritpitakphong et al. found the microbiome on the leaf surface of *A. thaliana* could protect the host against *Botrytis cinerea* and the key species might be *Pseudomonas* sp.^40^. Koskella et al.^41^ studied the relationship between bark-associated microbiota and horse chestnut bleeding canker disease, and found that the richness and Faith’s phylogenetic diversity in the symptomatic tree bark samples were lower than in the asymptomatic samples. Furthermore, there were significant negative correlations between the symptom index and the α-diversity^41^. In this study, the link between the microbiome of rapeseed stem and SSR was complicated. For bacterial communities, there were no significant differences in richness, evenness, and diversity among different tissues of the lesion. For fungal communities, the richness, evenness, and diversity in healthy tissues were significantly higher than in the diseased tissues. In nature, there is the niche differentiation between bacteria and fungi^42^. Compared to the competition with bacteria, the intraspecific competition of fungi might be more intense. As the dominant fungus in the diseased tissues, *S. sclerotiorum* consumed more resources and might inhibit other fungi. Moreover, the oxalic acid secreted by *S. sclerotiorum* could significantly decrease the host pH^43,44^. The sensitivity of bacteria and fungi to pH might be another reason. We also found that *Sclerotinia* existed not only in the diseased tissues but also in the healthy tissues with relatively high abundance. The higher diversity and balance of the microbiome might have been the reason that *Sclerotinia* could not cause lesion and damage plants in the healthy tissue.

At present, network theory has emerged as an extremely promising approach for modeling complex biological systems with multifaceted interactions between members, including microbiota, and will promote the applicability of the microbiome research to personalized medicine, public health, environmental and industrial applications, and agriculture^45^. It also provides opportunities to enhance disease management^46^. The connection and strength of the network are crucial for the resistance to the pathogens^23^. General features of many complex networks are scale-free, small-world, modular, and hierarchical^47^. In this study, we successfully constructed the scale-free, small-world, and modular networks. The nodes of the empirical networks of the treatment group were less than those of the control group, while the links were more. It meant that the bio-priming treatment promoted the interaction among microorganisms. In the network, there is an asymptotic negative linear relationship between the average path distance and global efficiency^48^. In our study, the average path distance of the treatment group network was significantly shorter than that of the control group, suggesting that the global efficiency of the treatment group network was higher than the control group. Based on the network topology calculated by within-module connectivity and connectivity among modules, hubs and connectors were defined as the keystone species^49,50^. Bio-priming treatment changed the amount and the kind of keystone species and the promotion of interaction between microorganisms was not independent on the number of hubs and connectors increased. Robustness is the measurement of the strength of the network^51^. The natural connectivity of the network in the treatment group was greater than that of the control group. In the face of random and targeted attacks, there was a smaller decrease in the natural connectivity in the treatment group network than in the control group network. It indicated greater network robustness and anti-disturbance ability of the treatment group interaction network was stronger. The stronger interaction network of microorganisms might inhibit the further extension of lesions and prevent lodging, thus protecting rapeseed from the further harm of SSR, increasing the tolerance for SSR, and reducing the yield loss. The results offer another explanation for the increased resistance of plants to phytopathogens after treated with BCAs and lay a theoretical foundation for plant disease management.

The occurrence and development of plant disease are in a dynamic and ongoing process, and plant pathogens are affected by other microorganisms in habitats. In networks, the taxa that have direct or indirect negative associations with the pathogen are potential candidates for biocontrol agents^46^. Among the 11 common genera, which had a direct negative interaction with *Sclerotinia*, we chose *Golubevia* and unidentified fungus 5 to verify by PCR and phylogenetic analysis. Unidentified fungus 5 (*E. linariae*) is a kind of plant pathogen and can cause the amphigenous leaf spot of *Plantaginaceae*, e.g., *Linaria genistifolia*, *Linaria repens*, and *Linaria vulgaris*^52^. *G. pallescens* is a basidiomycetous yeast^53^ and once belonged to *Tilletiopsis*^54^. *G. pallescens* could control rose and cucumber powdery mildew^55,56^. However, now the studies about the antagonistic interaction with *Sclerotinia* of these microorganisms are scarce. The hypothesis needs to be further confirmed with experiments.

Through many well-known mutualistic interactions between plant and microbiota, plant microbiome has been hardly considered in crop production strategies. To protect plants from plant diseases and control the yield loss, the effect on the plant microbiome could be one criterion of breeding new varieties, creating and finding new chemicals and BCAs. In the future, we will be able to make better use of the network theory and design a more robust plant microbiome to support plant health.

## Methods

### Rapeseeds bioprimed with *S. sclerotiorum* strain DT-8

*S. sclerotiorum* strain DT-8 carries a DNA virus SsHADV-1 and is a hypovirulent strain^13^. Rapeseeds (cv. Huashuang 4, a low erucic acid and low glucosinolate rapeseed cultivar, provided by the Institute of Rapeseed Genetics & Breeding, Huazhong Agricultural University) were surface sterilized with 2% sodium hypochlorite solution^57^ for 5 min, followed by three successive thorough rinses with sterilized distilled water (SDW). *S. sclerotiorum* strain DT-8 was shake-flask cultured in potato dextrose broth medium for 5 days at 20 °C, 200 r.p.m.; then, the *S. sclerotiorum* strain DT-8 hyphal fragment suspension was diluted to OD_600_ = 2.0 with SDW. Sterilized rapeseeds were soaked with the hyphal fragment suspension by 10 mL/5 g seeds. After 18 h of treatment at 20 °C, the seeds were air-dried to constant weight for pot trial and field experiment. Non-bioprimed seeds that soaked with SDW at 20 °C for 18 h were used as control.

### Field experiment

The field experiments were carried out in Huazhong Agricultural University (30°28′N, 114°21′E), Wuhan City, and Dongshan Village (30°20′N, E114°43′E), Ezhou City, Hubei Province, China, during 2016–2018. Rapeseed bioprimed with strain DT-8 was planted in blocks of 2 × 15 m (width × length) with non-bioprimed rapeseed as a control. Average of 720 rapeseed plants were planted in block naturally infected by *S. sclerotiorum*. At the flowering stage, the rapeseed was sprayed with fungicide prochloraz (150 g a.i./ha) or water as a control. All the field experiments were performed in a randomized block design and three replications were set for treatment and control. At the mature stage, the disease severity of SSR and the yield were measured. Disease severity was assessed at a 0–4 level according to Li et al.^58^. The control effect of SSR was calculated by disease severity. All data were tested with one-way analysis of variance, followed by Duncan’s new multiple range test (DMRT) (*P* < 0.05), with SPSS Statistics 19.0.0.

### Sample collection and DNA extraction

Non-bioprimed (Control, C) and bioprimed (Treatment, T) rapeseeds were sown in the experimental field in Zishi Town, Jingzhou City, Hubei Province, China (30°11′N, 112°24′E) on 28 September 2017. On 18 April 2018, at the stage of development of pod, the diseased stems of the control and treatment were randomly collected from the experimental fields by five-point sampling method. To control the variance of different morbidity degree of SSR and eliminate the effect of the low incidence of SSR of the bioprimed treatment, the stems with lesion more than 3 cm and less than 10 cm in diameter were retained. The diseased stem was divided into four parts, namely the center of the lesion (Part 1, P1), the edge of lesion (Part 2, P2), healthy tissue 1 (Part 3, P3), and healthy tissue 2 (Part 4, P4) (Supplementary Fig. 2a). Thus, we got eight kinds of samples named CP1, CP2, CP3, CP4 and TP1, TP2, TP3 and TP4. To study the impact of bio-priming treatment and SSR on rapeseed stem microbiome, we divided the samples into control group (CP1, CP2, CP3, CP4) and treatment group (TP1, TP2, TP3, TP4) according to whether rapeseeds were bioprimed, and divided the samples into the diseased group [Part 1 (CP1, TP1) and Part 2 (CP2, TP2)] and the healthy group [Part 3 (CP3, TP3) and Part 4 (CP4, TP4)] based on the different sampling parts. We pooled five stems that came from five sampling points, respectively, for each sample and the phloem and cortex of stems were collected and the genomic DNA was extracted. There are three replicates for each sample.

### PCR detection and sequencing

To test the SsHADV-1 in all the samples, the coat protein gene (*CP*) fragment of SsHADV-1 was amplified using specific primers (CP-F1: 5′-GGAGCATCCTCAACACGACAT C-3′ and CP-R1: 5′-TACGAAGAAGGTCGGACGCC-3′). Total volume of 25 μL PCR mixture contained 2.5 μL 10 × buffer (Mg^2+^) (New England Biolabs), 0.5 μL 10 mM dNTP mix (New England Biolabs), 0.5 μL of each primer (10 μM), 0.2 μL Taq DNA polymerase (5 U/μL) (New England Biolabs), 19.8 μL nuclease-free water, and 1 μL genomic DNA (10–100 ng). The conditions for PCR amplification included a denaturation step at 95 °C for 5 min and 30 cycles of 94 °C for 30 s, 58 °C for 45 s, and 72 °C for 5 min.

Using the genomic DNA as a template, the 16S rRNA gene and ITS fragment were amplified using specific primers (799F: 5′-AACMGGATTAGATACCCKG-3′ and 1193R: 5′-ACGTCATCCCCACCTTCC-3′ for 16S rRNA; ITS1-F: 5′-CTTGGTCATTTAGAGGAAGTAA-3′ and ITS2: 5′-GCTGCGTTCTTCATCGATGC-3′ for ITS)^59,60^ tagged with a barcode. PCR amplification was conducted in a total reaction volume of 30 μL containing 15 μL of Phusion High-Fidelity PCR Master Mix (New England Biolabs), 0.2 μM forward and reverse primers, and ~10 ng of template DNA. The thermal cycling consisted of initial denaturation at 98 °C for 1 min, followed by 30 denaturation cycles at 98 °C for 10 s, annealing at 50 °C for 30 s, elongation at 72 °C for 60 s, and finally 72 °C for 5 min. The PCR products were mixed in equal density ratios and purified using the Gene JET Gel Extraction Kit (Thermo Scientific). Sequencing libraries were generated using the NEB Next1 Ultra™ DNA Library Prep Kit for Illumina (NEB, USA) according to the manufacturer’s protocol and index codes were added. The DNA yield and quality of the library were assessed using the Qubit12.0 Fluorometer (Thermo Scientific) and the Agilent 2100 Bioanalyzer system. The library was sequenced on an Illumina HiSeq 2500 platform by Beijing Novo-gene Bioinformatics Technology Co. Ltd.

### Preprocessing and data analysis

Sequences were pre-processed, quality filtered, and analyzed by using Quantitative Insights into Microbial Ecology 2 (QIIME2) version 2018.6^61^. DADA2 software package was used to control the sequence quality and remove chimeras with the “consensus” method^62^. Taxonomic assignment of 16S rRNA gene and ITS fragments representative sequences was performed based on the Greengenes database^63^ and UNITE database^64^.

After discarding the no-target ASVs and low-abundance ASVs (<5 total counts)^65^, the rarefaction curves of 16S rRNA gene and ITS-sequencing data were created by QIIME2. To remove sample heterogeneity, the dataset was normalized to the lowest number of read counts (34,159 reads per 16S rRNA sequencing sample and 38,801 reads per ITS-sequencing sample) for further analysis. α-Diversity analysis was performed by QIIME2. For bacterial communities and fungal communities, the numbers of ASVs and Pielous’s evenness were used to evaluate the richness and evenness, respectively; Shannon’s diversity index and Faith’s phylogenetic diversity were used to calculate the diversity. Kruskal–Wallis test was used to analyze the statistical differences in α-diversity. β-Diversity was calculated by the weighted UniFrac distance with QIIME2^66^. PCoA was performed based on the weighted UniFrac distance matrix by R package vegan. The PERMANOVA^67^ with 999 random permutations was used to analyze statistical differences in β-diversity with QIIME2. The resulting *p*-values were adjusted for multiple comparisons using the Benjamini and Hochberg’s false discovery rate and an adjusted *P* < 0.05 (*q*-value < 0.05) was considered statistically significant^68^. LEfSe^69^ (http://huttenhower.sph.harvard.edu/galaxy) was used to elucidate the biomarker at the genus level with relative abundance above 0.01% between different groups. An α-significance level of 0.05 and an effect-size threshold of 2 were used for all biomarkers.

Possible plant pathogens were searched at the genus level according to Bull et al.^70^ and List of Plant Pathogenic Fungi 19 *Augus*, which were revised by the International Subcommission for the Taxonomy of Phytopathogenic Fungi at the International Commission on the Taxonomy of Fungi (https://www.fungaltaxonomy.org/files/2814/4052/7843/List_of_plant_pathogenic_fungi_19_August.doc).

Microbial interaction networks of the control and treatment groups were used to explore co-occurrence patterns of bacterial and fungal taxa. The genera of each group with relative abundances above 0.01% and occurring in >50% of all samples were selected. Molecular Ecological Network Analyses Pipeline (http://ieg4.rccc.ou.edu/mena) was used to create microbial interaction networks for the selected genera^71^. The relative abundance data were calculated by using the process described in a previous study^47^ and taken log-transferring before obtaining the Spearman’s correlation matrix. Then, the correlation matrix was converted to a similarity matrix. Subsequently, an adjacency matrix was derived from the similarity matrix by applying an appropriate threshold (*s*_t_), which was defined using the RMT-based network approach^49,71^. One hundred random networks were generated using the Maslov–Sneppen procedure^72^ to compare the network indices under different conditions. The Student’s *t*-test was employed to test the differences of network indices using the standard deviations derived from corresponding random networks (*P* < 0.05)^71^. We chose the small-world coefficient and natural connectivity to evaluate the small-world characteristic and robustness of network^73,74^. We also assessed the robustness of different networks to random and targeted attacks (node removals)^75^ using the natural connectivity, as a graph-theoretic measure of global network connectivity that reliably measures network robustness. We measured how the natural connectivity of the microbial network changed when nodes were sequentially removed from the network. We applied three different types of network attacks as follows: (I) random attack (number of simulations of random attack to be processed was 100), (II) targeted attack ordered by betweenness, and (III) targeted attack ordered by node degree. The greedy modularity optimization was used to module proceed separation and modularity calculation^76^. The connectivity of each node was determined based on its within-module connectivity (*Z*_*i*_) and among-module connectivity (*P*_*i*_)^77^. Node topologies were organized into four categories as follows: (I) module hubs (*Z*_*i*_ > 2.5, *P*_*i*_ ≤ 0.62); (II) network hubs (*Z*_*i*_ > 2.5, *P*_*i*_ > 0.62); (III) connectors (*Z*_*i*_ ≤ 2.5, *P*_*i*_ > 0.62); and (IV) peripherals (*Z*_*i*_ ≤ 2.5, *P*_*i*_ ≤ 0.62)^49,50^. Networks were visualized using the Cytoscape 3.7.0^78^.

### Verification of key interaction microorganism with *Sclerotinia* and phylogenetic analysis

*Golubevia* and unidentified fungus 5 were verified by PCR and phylogenetic analysis. According to the relative abundance, the representative sequences of highest relative abundance ASVs of *Golubevia* and unidentified fungus 5 were used to design the forward primer (GP: 5′-CACTTGTGAATCGTTGGAGCG-3′; UF: 5′-AACCTGCAGATGGATCATTA-3′). ITS of *Golubevia* and unidentified fungus 5 was amplified by GP/ITS4 (5′-TCCTCCGCTTATTGATATGC-3′) and UF/ITS4. PCR cycling conditions were 95 °C for 5 min followed by 30 cycles of 95 °C for 30 s, 53 °C for 30 s, 72 °C for 1 min followed by a final cycle of 72 °C for 5 min. PCR products were cloned into the pMD19-T vector using the pMD^TM^19-T Vector Cloning Kit (Takara Bio Inc.) and verified by PCR technique with vector-specific M13-F (5′-GTAAAACGACGGCCAGT-3′) and M13-R (5′-CAGGAAACAGCTATGAC-3′). Sequencing of independent clones for each fungus was performed on both strands using an automatic DNA sequencer. The sequences were used for phylogenetic analysis. Alignments were performed by Clustal W 2.0^79^ and phylogenetic trees were constructed by the neighbor-joining method with a bootstrap value of 1000 replicates in MEGA 7.0.26^80^.

### Reporting summary

Further information on experimental design is available in the Nature Research Reporting Summary linked to this paper.

## Supplementary information

Supplementary Information

Reporting Summary Checklist

## Data Availability

All raw data of 16S rRNA and ITS amplicon sequencing are available at Sequence Read Archive (SRP238718).

## Code availability

Our sequence processing workflow follows the official tutorials of QIIME2 (https://docs.qiime2.org/2018.6/tutorials/). Detailed parameters of PCoA and network attack analysis can be found in the Supplementary Note 1.
