# Supplementary material for: Bio-priming with a hypovirulent phytopathogenic fungus enhances the connection and strength of microbial interaction network in rapeseed
Source: NPJ Biofilms Microbiomes. 2020 Oct 30;6:45. doi: 10.1038/s41522-020-00157-5 (PMC7603479; doi:10.1038/s41522-020-00157-5)
Supplement: Supplementary file 1 — Supplementary Information [file 41522_2020_157_MOESM1_ESM.pdf]

Supplemental Material for:

Bio-priming with a hypovirulent phytopathogenic fungus enhances the connection and strength of microbial interaction network in rapeseed

Zheng Qu, Huizhang Zhao, Hongxiang Zhang, Qianqian Wang, Yao Yao, Jiasen Cheng, Yang Li, Jiatao Xie, Yanping Fu, Daohong Jiang

Table of Contents:

**Supplementary Table 1**	Page 2
**Supplementary Table 2**	Page 3
**Supplementary Table 3**	Page 4
**Supplementary Figure 1**	Page 5
**Supplementary Figure 2**	Page 6
**Supplementary Figure 3**	Page 7
**Supplementary Figure 4**	Page 8
**Supplementary Figure 5**	Page 9
**Supplementary Figure 6**	Page 10
**Supplementary Figure 7**	Page 11
**Supplementary References**	Page 12
**Supplementary Note 1**	Page 13

Supplementary Table 1 PERMANOVA on different parts

Microbial communities	Group 1	Group 2	pseudo-F	p-value	q-value
Bacterial communities	Part 1	Part 2	4.619	0.016	0.0192 *
		Part 3	44.632	0.003	0.006 **
		Part 4	41.754	0.005	0.0075 **
	Part 2	Part 3	12.479	0.002	0.006 **
		Part 4	10.92	0.002	0.006 **
	Part 3	Part 4	0.954	0.471	0.471
Fungal communities	Part 1	Part 2	1.893	0.173	0.173
		Part 3	42.497	0.001	0.006**
		Part 4	234.378	0.004	0.006 **
	Part 2	Part 3	42.93	0.003	0.006 **
		Part 4	246.794	0.003	0.006 **
	Part 3	Part 4	5.901	0.024	0.0288 *

The PERMANOVA with 999 random permutations was used to analyze statistical differences between different parts in beta diversity. Levels of significance: * q-value<0.05, ** q-value<0.01

Supplementary Table 2 Topological properties of the random networks of microbial communities

Condition	Average path distance	Avg clustering coefficient	Modularity
Control	3.131 +/- 0.064	0.075 +/- 0.021	0.430 +/- 0.015
Treatment	2.810 +/- 0.053	0.116 +/- 0.016	0.344 +/- 0.010

Supplementary Table 3 Diseases of rapeseed

Category	Disease	Pathogen	Reference
Bacterial diseases	Bacterial black rot	*Xanthomonas*	1
	Bacterial leaf spot	*Xanthomonas*	2
	Bacterial pod rot	*Pseudomonas*	2
	Bacterial wilt and Rot	*Erwinia*	1
	Scab	*Streptomyces*	2
	Crown gall	*Agrobacterium*	3
Fungal diseases	Alternaria black spot	*Alternaria*	4
	Seed rot, damping-off	*Fusarium*<br>*Gliocladium*	2
	Foot rot	*Fusarium*	1
	Leaf spot	*Alternaria*	5
	Pod drop	*Cladosporium*<br>*Alternaria*	6
	Sclerotinia stem rot	*Sclerotinia*	7
	White leaf spot and gray stem<br>Ring spot	*Mycosphaerella*	1, 8
	Verticillium wilt	*Verticillium*	9

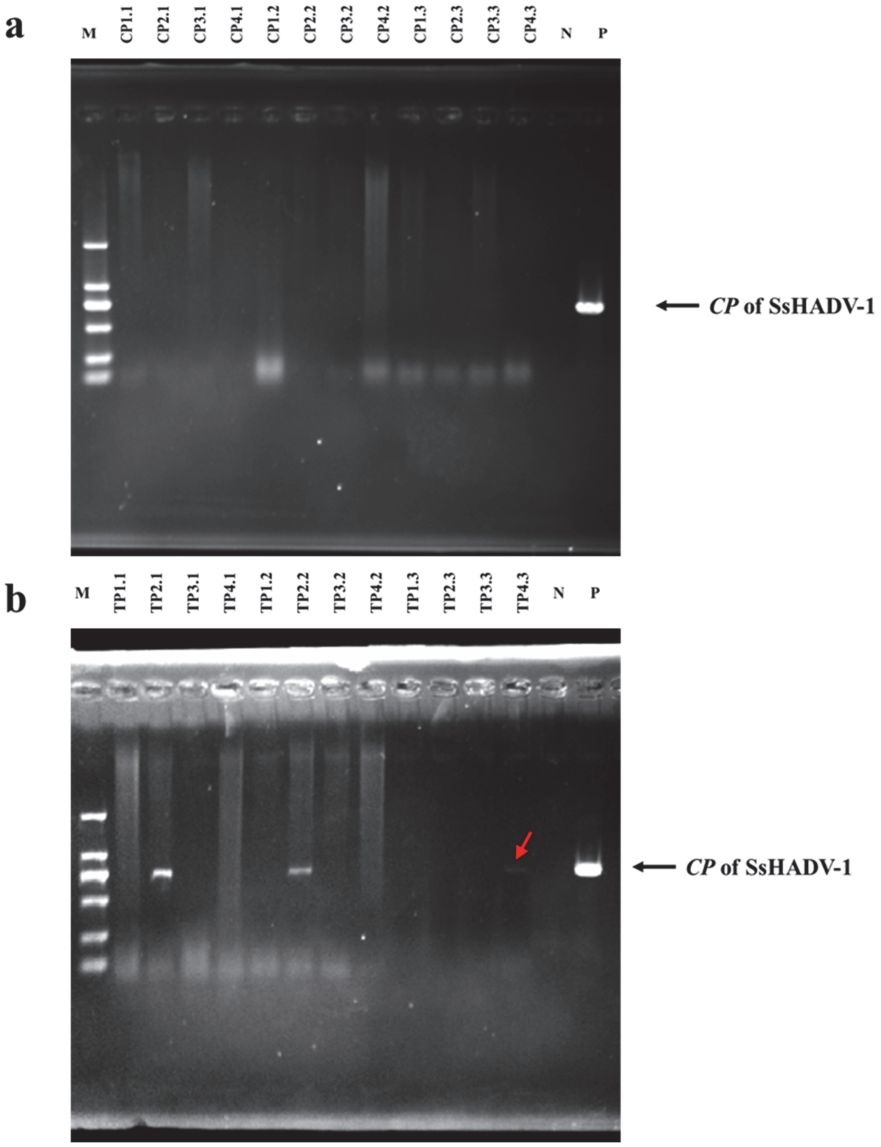

Supplementary Figure 1 The PCR test of the SsHADV-1 in different samples. **a** Samples of *S. sclerotiorum*-infected rapeseed stem of the non-biopriming treatment. **b** Samples of *S. sclerotiorum*-infected rapeseed stem of the bio-priming treatment. Lane M: the DL2000 DNA Maker. Lane N: Negative control (H_2O). Lane P: Positive control (The genome DNA of *S. sclerotiorum* DT-8). Samples are derived from the same experiments and processed in parallel.

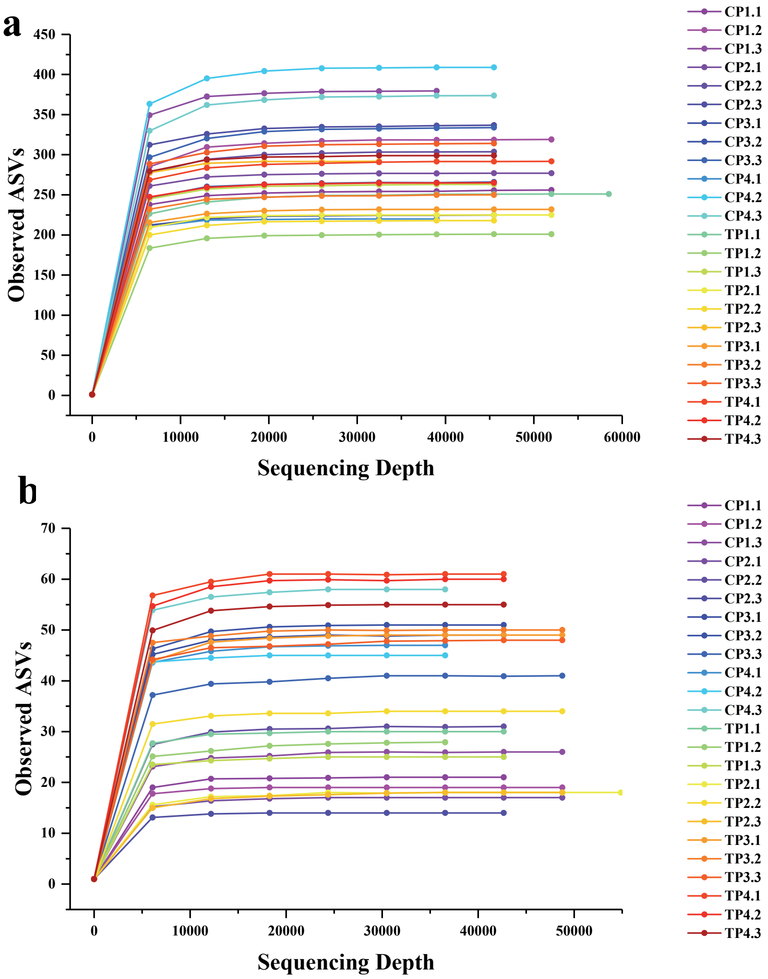

Supplementary Figure 2 The rarefaction curves of 16S rRNA sequencing samples and ITS sequencing samples. **a** The rarefaction curves of 16S rRNA sequencing samples. **b** The rarefaction curves of ITS sequencing samples.

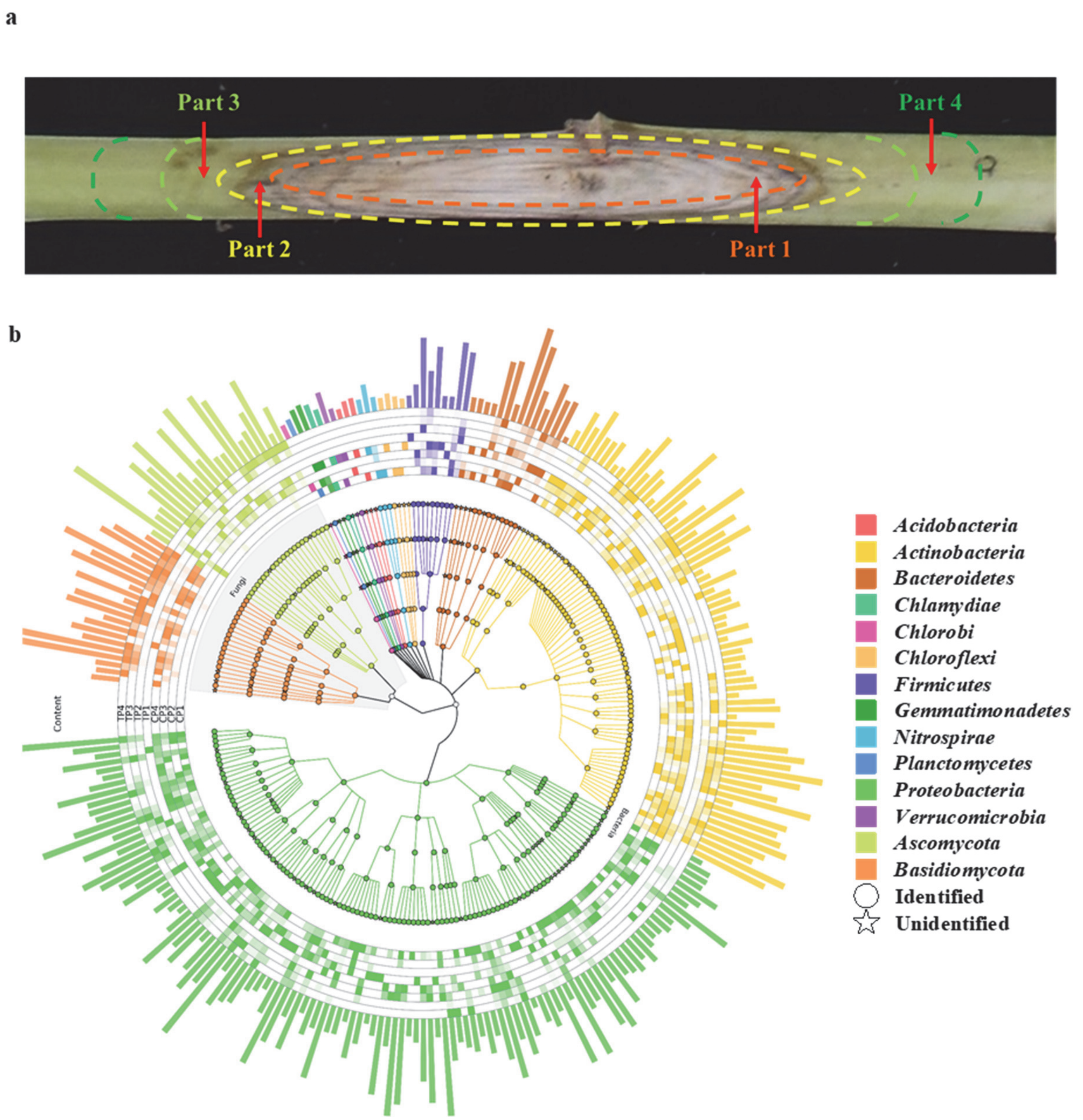

Supplementary Figure 3 The four parts of rapeseed diseased stems and biodiversity of bacterial and fungal communities. **a** The four parts of natural diseased rapeseed stem. **b** The biodiversity of bacterial and fungal communities.

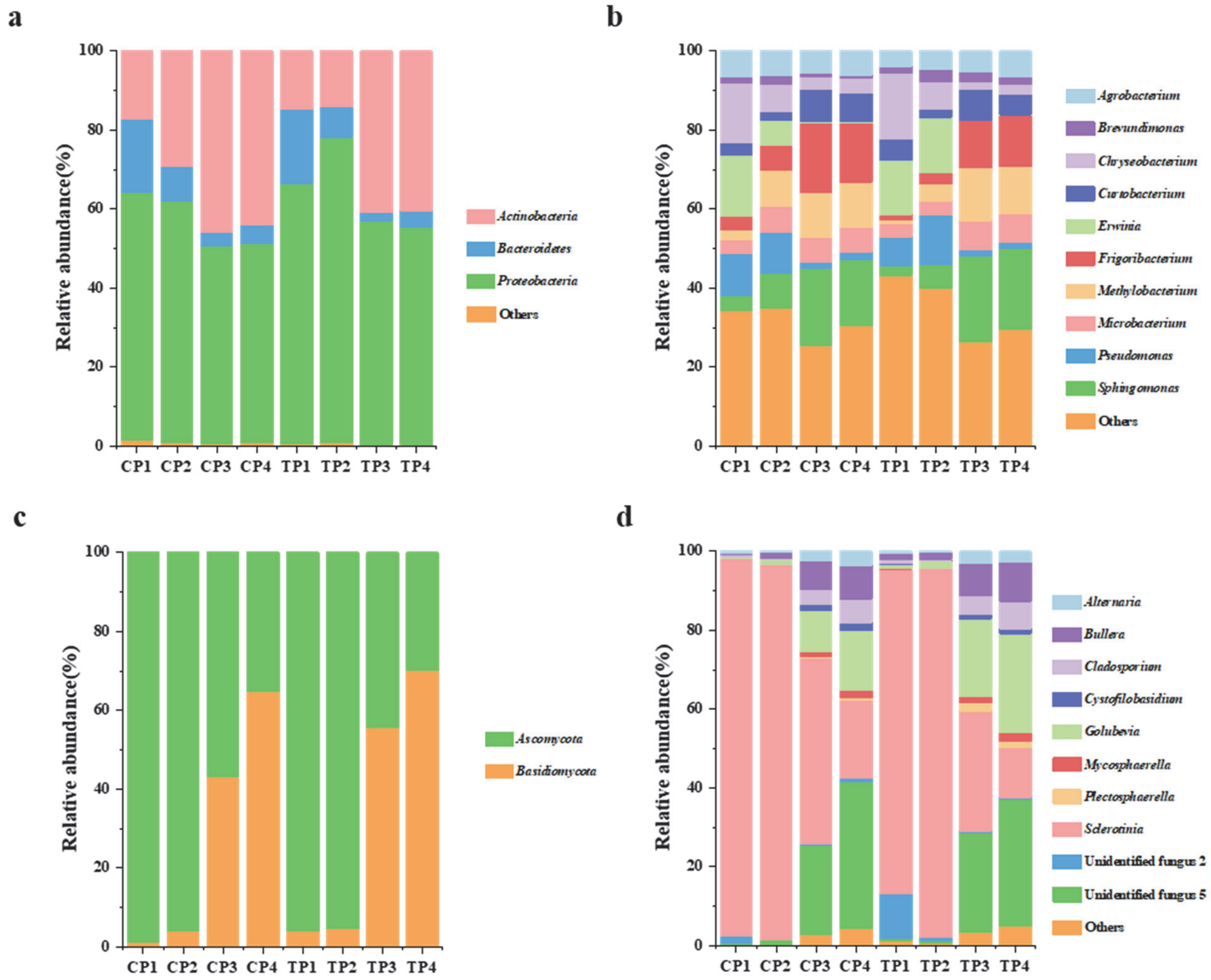

Supplementary Figure 4 The composition of bacterial and fungal communities. **a** The bacterial communities composition at the phylum level. **b** The bacterial communities composition at the genus level. **c** The fungal communities composition at the phylum level. **d** The fungal communities composition at the genus level.

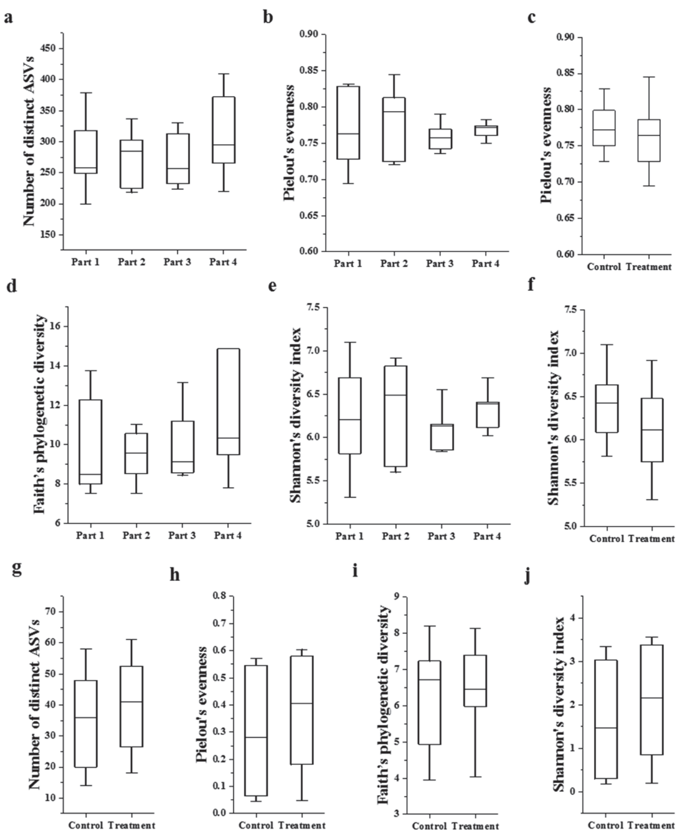

Supplementary Figure 5 The alpha diversity of bacterial and fungal communities. **a** Number of distinct ASVs bacterial communities in different parts. **b** The Pielous's evenness of bacterial communities in different parts. **c** The Pielous's evenness of bacterial communities in different treatments. **d** The Faith's phylogenetic diversity of bacterial communities in different parts. **e** The Shannon's diversity index of bacterial communities in different parts. **f** The Shannon's diversity index of bacterial communities in different treatments. **g** Number of distinct ASVs fungal communities in different treatments. **h** The Pielous's evenness of fungal communities in different treatments. **i** The Faith's phylogenetic diversity of fungal communities in different treatments. **j** The Shannon's diversity index of fungal communities in different treatments. The Kruskal-Wallis test was used to analyze the statistical differences in alpha diversity.

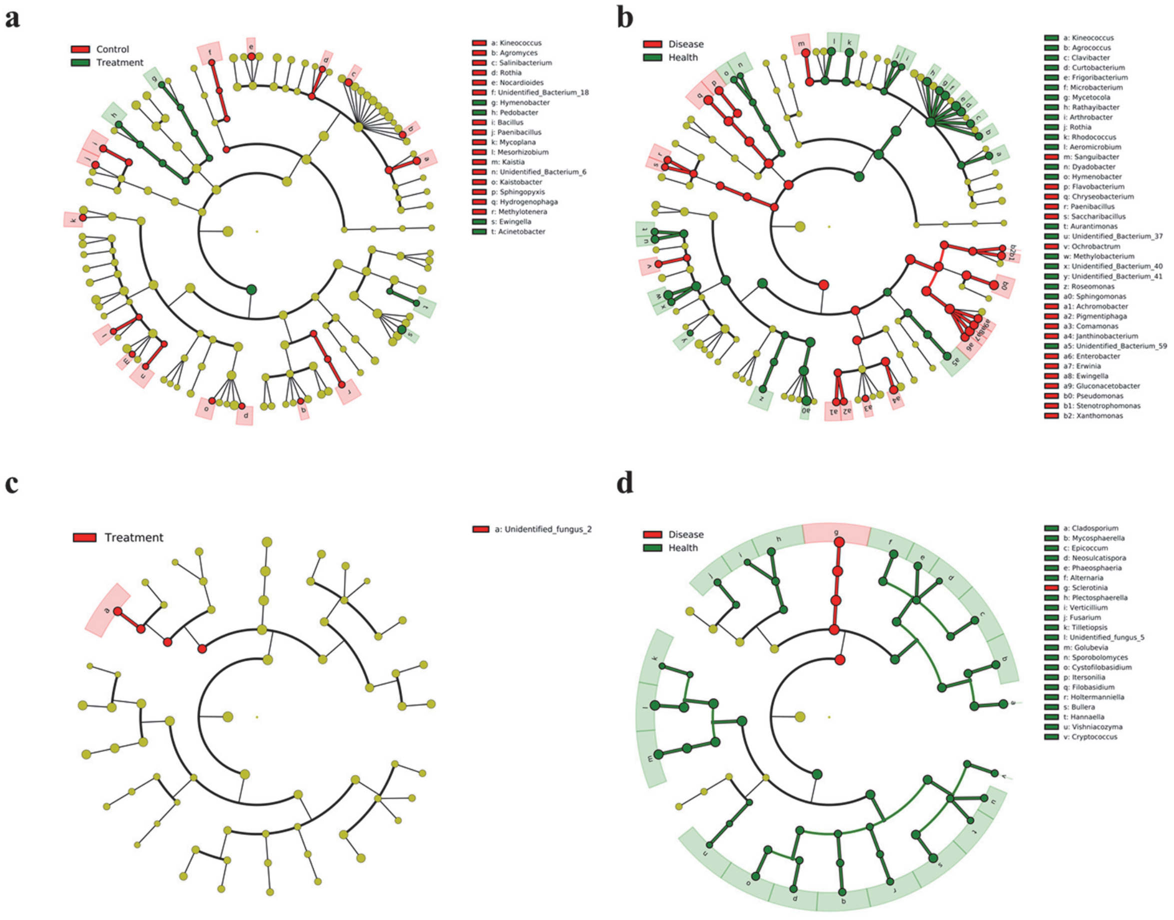

Supplementary Figure 6 LEfSe analysis of microbial communities between different groups at the genus level. **a** The biomarkers of bacterial communities between the control and treatment groups. **b** The biomarkers of bacterial communities between the diseased and healthy groups. **c** The biomarkers of fungal communities between the control and treatment groups. **d** The biomarkers of fungal communities between the diseased and healthy groups.

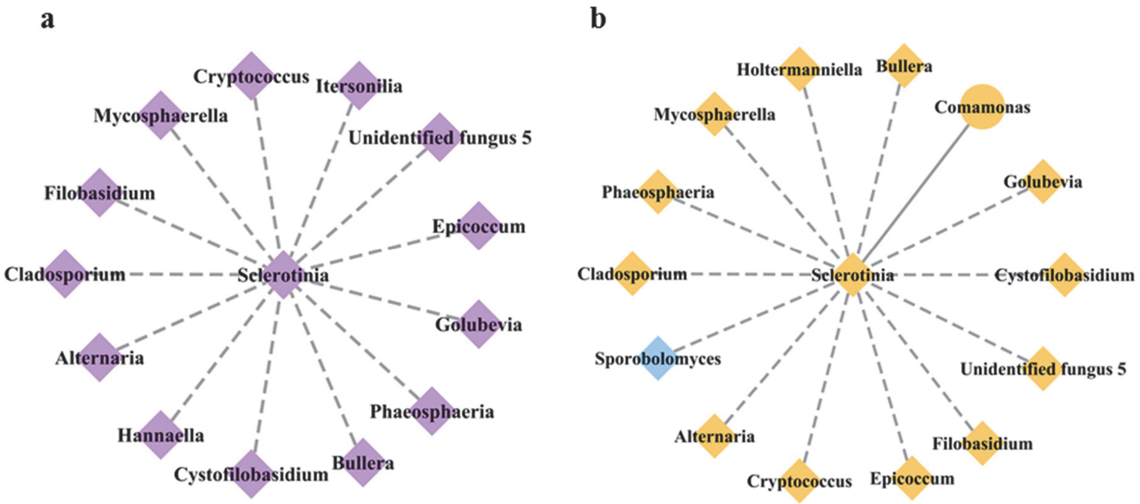

Supplementary Figure 7 The microorganisms which had direct interactions with *Sclerotinia* at the genus level. **a** The microorganisms which had direct interactions with *Sclerotinia* in the control group. **b** The microorganisms which had direct interactions with *Sclerotinia* in the treatment group.

Supplementary References

1. Nyvall, R.F. Field crop diseases handbook. (Springer Science & Business Media, New York; 2013).
2. Kharbanda, P.D. et al. Diseases of Rapeseed = Canola (*B. napus* L. and *Brassica rapa* L.(= *B. campestris* L.)), https://www.apsnet.org/edcenter/resources/commonnames/Pages/Rapeseed.aspx,(2001).
3. Charest, P.J., Iyer, V. & Miki, B.L. Virulence of *Agrobacterium tumefaciens* strains with *Brassica napus* and *Brassica juncea*. *Plant Cell Rep.* **8**, 303-306 (1989).
4. Conn, K.L., Tewari, J.P. & Awasthi, R.P. A disease assessment key for *Alternaria* blackspot in rapeseed and mustard. *Can. Plant Dis. Surv.* **70**, 19 (1990).
5. Ram, R. & Chauhan, V. Assessment of yield losses due to *Alternaria* leaf spot in various cultivars of mustard and rapeseed. *J. Mycopathol. Res.* **36**, 109-111 (1998).
6. Pétrie, G.A. Diseases of *Brassica species* in Saskatchewan, 1970-72 Ⅱ. Stem, pod, and leaf spots. *Can. Plant Dis. Surv.* **53**, 83-87 (1973).
7. Boland., G. & Hall., R. Index of plant hosts of *Sclerotinia sclerotiorum*. *Can. J. Plant Pathol.* **16**, 93-108 (1994).
8. Berkenkamp, B. & Degenhardt, K. Diseases of rapeseed in central and northern Alberta in 1971. *Can. Plant Dis. Surv.* **52**, 62-63 (1972).
9. Heale, J.B. & Karapapa, V.K. The verticillium threat to Canada's major oilseed crop: canola. *Can. J. Plant Pathol.* **21**, 1-7 (1999).

Supplementary Note 1: R workflow of the PCoA and network attack analysis.

```
#PCoA

#Libraries
library("vegan")

#Load metadata
group_data = read.table("Metadata.txt", sep="\t", header=T, check.names=F, comment.char = "",row.names=1)

#Load Distance matrix
expr = read.table("weighted_unifrac-distance-matrix.txt", row.names=1, sep="\t", header=T, check.names = F)

#Performing PCoA
samples_name <- colnames(expr)
groups_sample <- row.names(group_data)
used <- samples_name %in% groups_sample
expr <- expr[, groups_sample]
pcoa <- cmdscale(expr, k=3, eig=T)
```

```
points <- as.data.frame(pcoa$points)

colnames(points) <- c("x", "y", "z")

eig <- pcoa$eig

PcoA1 <- -eig[1] / sum(eig)

PcoA2 <- -eig[2] / sum(eig)

points <- cbind(points, expr[match(rownames(points), rownames(expr)), ])


# Results output

write.table(points,file="PCoA.txt",sep="\t",quote=F,row.names = T,col.names = T)


#Network attack analysis


#Libraries

library(SpiecEasi)

require(reshape2)

library(phyloseq)

library(igraph)


#Calculating the natural connectivity from adjacency matrix

ncc <- function(ig) {

  evals <- eigen(ig)$value
```

```
    nc <- log(mean(exp(evals)))
}

#Calculating the natural connectivity from adjacency matrix of a graph
natcon <- function(ig) {
    adj <- get.adjacency(ig)
    evals <- eigen(adj)$value
    nc <- log(mean(exp(evals)))
}

#Targeted attack ordered by betweenness
nc.attackbetweenness <- function(ig) {
    hubord <- order(rank(betweenness(ig)), decreasing=TRUE)
    sapply(1:round(vcount(ig)*.8), function(i) {
        ind <- hubord[1:i]
        tmp <- delete_vertices(ig, V(ig)$name[ind])
        natcon(tmp)
    })
}

#Targeted attack ordered by node degree.
```

```
nc.attackdegree <- function(ig) {
  hubord <- order(rank(degree(ig)), decreasing=TRUE)
  sapply(1:round(vcount(ig)*.8), function(i) {
    ind <- hubord[1:i]
    tmp <- delete_vertices(ig, V(ig)$name[ind])
    natcon(tmp)
  })
}

#Node removals
attack<-function (adj.mat, node.sup)
{
  n.nodes <- dim(adj.mat)[1]
  adj.mat[node.sup, ] <- rep(0, n.nodes)
  adj.mat[, node.sup] <- rep(0, n.nodes)
  nc<-ncc(adj.mat)
  list(new.mat = adj.mat, nc=nc)
}

#Random attack
random<-function (adj.mat, max.remove, nsim)
```

```
{
  a<-adj.mat
  n.nodes <- dim(adj.mat)[1]
  mean.nc<-rep(0, n.nodes)
  for (i in 1:nsim) {
    remove <- rep(0, n.nodes)
    rem.nodes <- rep(0, n.nodes)
    nc<-rep(0, n.nodes)
    for (j in 1:max.remove) {
      k <- 0
      tmp <- ceiling(runif(1, 0, (n.nodes - j + 1)))
      while (tmp > 0) {
        k <- k + 1
        if (remove[k] == 0)
          tmp <- tmp - 1
      }
      remove[k] <- 1
      rem.nodes[j] <- k
      rm <- attack(adj.mat, k)
      adj.mat <- rm$new.mat
      nc[j] <- rm$nc
```

```
        }
        mean.nc <- mean.nc+nc
        adj.mat<-a
    }
    mean.nc <- mean.nc/nsim
    list(new.mat = adj.mat,mean.nc=mean.nc,nc=nc,rem.nodes=rem.nodes)
}

#Load adjacent matrix data
data <- read.table(' adjacent matrix .txt', row.names=1,header =T,stringsAsFactors = F,sep = '\t')

#Data transformation
mdata <- as.matrix(data)
gra.data <- adj2igraph(mdata)

#Network attack analysis
nc.deg <- nc.attackdegree (gra.data)
nc.bet <- nc.attackbetweenness (gra.data)
nc.ran<-random(mdata,80,100)

#Results output
```

```
write.table(nc.deg,file="deg-att.txt",sep="\t",quote=F,row.names = T,col.names = T)

write.table(nc.bet,file="bet-att.txt",sep="\t",quote=F,row.names = T,col.names = T)

write.table(nc.ran$mean.nc,file="ran-att.txt",sep="\t",quote=F,row.names = T,col.names = T)
```
